# Supplementary material for: EXAM: Ex-vivo allograft monitoring dashboard for the analysis of hypothermic machine perfusion data in deceased-donor kidney transplantation
Source: PLOS Digit Health. 2024 Dec 18;3(12):e0000691. doi: 10.1371/journal.pdig.0000691 (PMC11654979; doi:10.1371/journal.pdig.0000691)
Supplement: Attachment — Submitted filename: EXAM-Plos DH-response-final.docx [file pdig.0000691.s001.docx]

Response to reviewer comments

**Title:** EXAM: Ex vivo allograft monitoring dashboard for the analysis of hypothermic machine perfusion data in deceased-donor kidney transplantation

**Manuscript Number:** PDIG-D-24-00069

Responses by Simon Schwab on behalf of all the co-authors.

# 0. Editor

**Journal Requirements:**

*1. Please update your online Competing Interests statement. If you have no competing interests to declare, please state: “The authors have declared that no competing interests exist.”*

I have updated the statement in the manuscript and the submission system.

*2. Please provide separate figure files in .tif or .eps format only and remove any figures embedded in your manuscript file. Please also ensure that all files are under our size limit of 10MB. You may leave the figure captions or legends in the manuscript.*

I have uploaded the figures as TIF files and removed the figures from the manuscript while keeping the legend.

*3. Potential Copyright Issues: Your manuscript (header) and Figure 3 contain branding/a logo. We are not permitted to publish this under our CC-BY 4.0 license, even with permission. We ask that you please remove or replace it.*

I have removed the Swisstransplant logo from the manuscript header and Figure 3.

# Reviewer Responses to Questions

*1. Does this manuscript meet PLOS Digital Health’s publication criteria? Is the manuscript technically sound, and do the data support the conclusions? The manuscript must describe methodologically and ethically rigorous research with conclusions that are appropriately drawn based on the data presented.*

*Reviewer #1: No*

*Reviewer #2: Partly*

*Reviewer #3: Partly*

We acknowledge the objections that our work only partly meets the publication criteria and hope to clarify below. The six PLOS Digital Health criteria are as follows:

1. **Originality:** We developed the tool EXAM entirely from scratch, as thoroughly documented in the GitHub repositories referenced in the manuscript. Therefore, this is unequivocally original work conducted solely by us, providing a highly valuable tool for transplant organizations and centers worldwide.
2. **High importance and broad interest to community of researchers, engineers and clinicians working in the field of digital health:** The target community is kidney transplantation professionals and transplant organizations. Worldwide there were over 62,000 kidney transplantations from deceased donors reported in 2022 (data based on the Global Observatory on Donation and Transplantation (GODT) data, produced by the WHO-ONT collaboration). An increasing part of those procured kidneys are preserved, reconditioned and assessed by ex-vivo machine perfusion. Generally, digital tools to facilitate processes are of great importance in the transplant community because of the medical urgency of the intervention. Specifically, EXAM is important to Swisstransplant which, like other transplant organizations, is obliged to review kidney perfusion data and perform quality control. From our experience in Switzerland, we know that kidney transplant surgeons may review the data before transplantation. For example, when organ resistance is very high, as indicated by the machine, kidney transplant surgeons would find it extremely helpful to look at the complete time series to have additional information for their clinical decision-making. Thus, three Swiss transplant centers were closely involved in this work as well and are using EXAM already. The EXAM online viewer enables everyone to quickly examine perfusion data from any device with an internet connection.
3. **High methodological rigor and ethical standards:** The tool has been carefully validated to read data correctly (time series, device and organ information such as the device's serial number, Unit ID, Start time, etc.). Validation is really straightforward by comparing EXAM with the original case report PDFs, which are created by software from ORS (ORS Data Station), the manufacturer of the LifePort perfusion machine. We made a case report available, and if reviewers wish to do so, they can compare the case report with the EXAM online viewer; see Readme <https://github.com/Swisstransplant/EXAM>, section “Validation.” Furthermore, we followed ethical standards by submitting the project to the local ethics committee, which determined it exempt from review and informed consent (KEK Bern; 2023-00557).
4. **Substantial evidence for its conclusions:** We conclude that EXAM is a valuable tool for viewing perfusion data and ensuring quality assurance. As an operative organization, our experience in designing and utilizing EXAM demonstrates its effectiveness. We encourage others to verify its usefulness through the live dashboard.
5. **Clearly outlined utility and accessibility for the broader community:** We provide the live dashboard for free and have decided to make EXAM open source and available on GitHub so it can be shared and developed by the broader community. We hope this tool will prove useful for other transplant organizations as well.
6. **Follow appropriate standards and practice of open science:** The main outcome of this work is the EXAM software. I have already outlined that EXAM is open source and available on GitHub.

*2. Has the statistical analysis been performed appropriately and rigorously?*

*Reviewer #1: No*

*Reviewer #2: No*

*Reviewer #3: No*

The reviewers believe that the statistical analysis was not appropriate. However, in their comments below, they do not raise any issues with the statistical analysis. As certified statistician (RSS GradStat), I would like to assure the editor that the statistical analysis is very straightforward as only descriptives are reported; the indicators are means, medians, standard deviations, or perfusion durations, and we show their distributions using histograms. Likewise the signal filtering of the time series is very standard by using a moving average, and I have illustrated this in Figure 2. Thus, the reviewers' conclusions may have little substance when no specific comments are provided, and we believe that they rather reflect the fact that no inferential statistics were required for this work (e.g., *p*-values).

*3. Have the authors made all data underlying the findings in their manuscript fully available (please refer to the Data Availability Statement at the start of the manuscript PDF file)?*

*The PLOS Data policy requires authors to make all data underlying the findings described in their manuscript fully available without restriction, with rare exception. The data should be provided as part of the manuscript or its supporting information, or deposited to a public repository. For example, in addition to summary statistics, the data points behind means, medians and variance measures should be available. If there are restrictions on publicly sharing data—e.g. participant privacy or use of data from a third party—those must be specified.*

*Reviewer #1: No*

*Reviewer #2: No*

*Reviewer #3: No*

We fully understand the reviewers' concern that we did not share the data. It is important to recognize that the main result of this work is the EXAM software, which is fully open-source and available. We also made two anonymized real datasets available as examples and part of EXAM. The aim of the EXAM tool is to examine *individual* cases. Sharing the complete dataset would require anonymizing all the data, particularly the transplant date. We don’t see how this would add to the publication as no conclusions are drawn from the complete dataset and no inference on a population is made. We only report descriptives and show histograms.

However, please let us know if the editor follows the reviewers' assessment and insists that we share the data. We believe our work would align with the PLOS policy above that requires sharing “data underlying the findings.” We kindly ask the Editor to give us advice on this issue.

*4. Is the manuscript presented in an intelligible fashion and written in standard English?*

*PLOS Digital Health does not copyedit accepted manuscripts, so the language in submitted articles must be clear, correct, and unambiguous. Any typographical or grammatical errors should be corrected at revision, so please note any specific errors here.*

*Reviewer #1: No*

*Reviewer #2: Yes*

*Reviewer #3: No*

We understand that two reviewers do not appreciate the quality of the writing. We apologized and improved the language of the manuscript with a professional service.

# Review Comments to the Author

## Reviewer #1

*1.1 Write a separate section for literature review/ Related work*

This work presents a tool to examine data from the LifePort machine. To the best of our knowledge, there is no R package to read and analyze data from the LifePort perfusion machine, so we created it in the first place. If the reviewer knows specific work relevant to the present manuscript, please let us know.

*1.2 Research requires mathematical & statistical analysis. But no mathematical equations found in the study.*

We don’t believe this is necessary. The analysis is only descriptive statistics; the indicators are just sample means, medians, standard deviations, and durations.

*1.3 Draw a work flow diagram to implement the methodology.*

The methodology includes three steps: data import, data processing, and calculating indicators. This is highlighted in Table 1 with details; we hope the reviewer finds this useful. We have improved the Table legend as follows:

Summary of main functions and procedures for processing LifePort HMP data. The data is processed in three steps: data import, data processing, and calculation of the statistical indicators.

*1.4 Write an algorithm to support the methodology*

We do not understand this suggestion and how it would improve our work.

*1.5 Need a comparative study with results with this study and related similar types*

*already published articles. (In tabular form)*

To the best of our knowledge, there are no similar software publications for machine perfusion data. We used the following PubMed query:

(dashboard[tiab] OR software[tiab] OR "R package"[tiab] OR Python[tiab]) AND "machine perfusion"[tiab] AND "transplantation"

We found only one publication, but it was related to optical imaging during machine perfusion as an additional source of clinical information. Thus, a comparative table with similar types of studies would not be necessary. It would be useful if there were a number of tools for the same aim (analyze and visualize machine perfusion data), but there are none. This is why developed EXAM in the first place, and this is why it is so valuable.

*1.6 Need few more references from recent publications.*

We would appreciate it if the reviewer could be more specific; we do not think it is necessary.

## Reviewer #2

*2.1. The topic of the paper is interesting but it needs major improvements as below. The abstracts should be rewritten to show more results and what was achieved from the created dashboard. The present abstract is very brief and only highlights the background and methods with minimal findings.*

Thank you very much for this helpful comment. We have improved the abstract and now report the main results and improved the conclusions:

We explain how data were processed and indicators were calculated, and we present summary statistics for N=255 kidney allografts receiving machine perfusion in Switzerland between 2020 and 2023. Median (interdecile range, IDR) of the main indicators were as follows: perfusion duration 5.18 hours (2.29−11.2), flow rate 110 ml/min (52.9−167), ice temperature 1.97°C (1.53−3.07), and perfusate temperature 6.68°C (5.58−8.36). We implemented the dashboard to identify issues, such as atypical perfusion parameters, high ice, or high perfusate tempera-ture to inform transplant centers for quality assurance. In conclusion, EXAM is a free tool that statisticians and data scientists can quickly deploy to enable quality control at transplant organ-izations that use LifePort kidney transporters. An online viewer is available at https://swisstransplant.shinyapps.io/EXAM/.

*2.2. Remove R programming language from the keywords.*

We removed all the keywords as they are not required by the journal.

*2.3. In the method section, it is not clear whether the data collected were anonymous. Was there a framework for data collection?*

We agree with the reviewer and clarified our data collection in a new subsection “Data collection”, see Section Materials and Methods:

Coded data from deceased kidney donors were collected at the six kidney transplant centers in Switzerland (Inselspital, Bern University Hospital; University Hospital of Geneva; Cantonal Hospital St. Gallen; University Hospital Basel; Lausanne University Hospital; University Hospital Zurich). Each center was equipped with two LifePort perfusion machines (Organ Recovery Systems; ORS).

The data collection was not anonymous. The data was coded, i.e., the hospital had to enter the deceased donor identifier in the LifePort machine. The machine was then sent to the transplant center of the kidney recipient, which then performed the surgery.

*2.4. What was done about missing data?*

In the first paragraph of the result section, we reported missing data, the cause of missingness, and that these cases were excluded; the section contains the following information:

We collected 266 LifePort datasets from 1. January 2020 to 31. December 2023. Eleven cases (4.1%) were excluded due to missing data in calculating the statistical indicators. These were caused by a perfusion time of below 30 minutes, and in such cases, flow and vascular resistance indicators were not calculated. Thus, the analysis data set had a sample size of N=255.

We would like to mention that we only had very little missing data (less than 5%), so we performed a complete case analysis for the descriptives in Table 3 and the histograms in Table 3. In quality assurance, however, missing data is not a concern but can be highly informative. For example, a missing mean vascular resistance and flow rate value indicates that machine perfusion failed or that the perfusion was too short to calculate the indicator (i.e., overall perfusion duration was below 30 minutes).

*2.5. How were the findings validated?*

Thank you for this question. The validation was very straightforward.

The time series data were directly from the raw data file, and only minimal processing (smoothing) was performed. These time series can be compared to the figures from the ORS case report (a PDF generated by the software from the LifePort manufacturer). We have made an example available; see Readme, section “Validation” (<https://github.com/Swisstransplant/EXAM>). It is possible to compare the PDF report to the data in the live dashboard (ST-0001-Example), and we have compared numerous cases to ensure the visualizations were accurate.

The indicators are only summary statistics, such as means, standard deviations, durations, etc. We do not think they require validation. The interactive figures in the dashboard can show single values of the time series, which facilitates comparing them to the summaries from the statistical indicators.

We added the following subsection “Validation” at the end of the Materials and Methods section:

We validated the data displayed by the EXAM dashboard (time series) by comparing our figures with the case reports created by the manufacturer's software (ORS Data Station). However, we directly read the time series data from the raw data files with minimal processing (smoothing). The indicators are based on medians, means, or standard deviations and do not require validation.

*2.6. The parameters collected seem to be incomprehensive. For example patients' clinical characteristics such as comorbidities, risk factors etc... should be included.*

Thank you for this comment; we added some basic deceased-donor characteristics at the beginning of the Results section:

The analysis data set had a sample size of N=255 kidneys with machine perfusion. These kidneys were from 184 deceased donors, of which 131 (71.2%) were organ donations after circulatory death (DCD), and 53 (28.8%) were donations after brain death (DBD). The median age of the donors was 58 years (range 11–86 years).

This work focused on kidney allografts rather than patients to ensure the quality of the HMP intervention: EXAM enables a quality assessment to ensure that each kidney receives optimal treatment in terms of perfusion and temperature indicators, regardless of the deceased donor or recipient's characteristics. Therefore, we believe that more specific clinical information about donors and recipients may not be particularly helpful.

*2.7. In the results, it is not clear how these indicators helped the patients.*

The EXAM dashboard can assess the quality of machine perfusion of explanted kidneys from deceased organ donors. The effectiveness of the treatment (machine perfusion) is based on evidence from randomized controlled trials (RCTs) and systematic reviews mentioned in the Introduction. However, if temperature and perfusion parameters are not optimal, the machine may automatically halt the perfusion. In such situations, the treatment loses its benefit, reducing it to standard care (cold storage). We have highlighted this in the Discussion section:

EXAM was designed for retrospective analysis of HMP data for quality control purposes to help identify problems to improve and optimize kidney HMP. Kidney allografts, mainly marginal kidneys, should receive the best treatment in the critical period between procurement and transplantation. This may enhance patient outcomes and help reduce additional costs, for example, when fewer kidney recipients experience delayed graft function after transplantation.

Furthermore, we explained in the Author's Summary how the tool can help patients:

Our work will enable transplant organizations to inspect their data, perform statistics and quality checks to help identify potential problems, and optimize the intervention to the benefit of kidney recipients.

In other words, we cannot just put the kidney in a perfusion machine and hope for the best. The perfusion parameters need to be in the optimal range; thus, it is worth investigating this data for quality control purposes.

*2.8. The discussion is rather a conclusion that is under-referenced. The discussion section is too brief and does not justify the findings in terms of the literature. It should be rewritten to highlight the implications of the findings on the literature and on practice*

Thank you. We have added a few more aspects in the Discussion section, first a reiteration of the clinical importance of HMP and a section on limitations; the two paragraphs are copied below. However, we would find it much more helpful if the reviewer was more specific about what was missing and why there should be more references. To the best of our knowledge, there simply are no publications describing similar HMP data processing.

There is ample evidence that HMP is effectively lower DGF and prolongs graft survival [3,4]. However, this requires optimal conditions in terms of perfusion and temperature during the treatment of the kidney allograft. One criticism of clinical trials is that they are artificial and do not provide real-life evidence. Therefore, quality assurance of the intervention in clinical practice n is crucial to achieving the treatment benefit.

A limitation of the study may be the risk in case the manufacturer ORS changes the structure of the data file. Then, EXAM would not work anymore with new data. However, the risk is largely mitigated as the tool is under an open-source license. This would enable everyone, not only Swisstransplant, to modify and share a new version of the tool as long as it is released under the same conditions, i.e., everyone can again modify and share the code, as stated in the GPL/AGPL licenses.

*2.9. The conclusion should be more summative and informative*

We have improved the conclusions as follows:

EXAM is a dashboard based on R and Quarto to visualize HMP data from the LifePort kidney transporter. Quality control with EXAM can identify problems during HMP and support guidelines, checklists, and training for healthcare providers. This assures optimal therapy, which has been demonstrated to be effective in preventing DGF and dialysis after transplantation.

## Reviewer #3

*Abstract and Author Summary*

*3.1 What is not clear from the abstract and author summary is whether EXAM is a new tool that the authors have developed. The authors say there is currently no free software. Then immediately talk about R which is an open source software. Please clarify this.*

We thank the reviewer for the opportunity to clarify this. EXAM is a new tool that we developed. We clarified this by changing the wording in the abstract from “implemented” to “developed” and from “the backbone is a collection of functions in the R programming language” to “we wrote functions in the R programming language”. The GitHub repository is proof that this is the case.

*Introduction*

*3.2 There same issue with abstract and author summary. EXAM is being introduced without clear information whether this is an existing platform, is it an R package? Did the authors develop it from scratch? Please clarify.*

Thank you for this comment. Yes, we developed it using the statistical programming language R. To clarify this, we edited the Abstract, Author Summary, and the last paragraph in the introduction.

*3.3 There is a hanging statement in paragraph three… There are two significant ??*

Thank you for spotting this; we removed this part.

*Development and Implementation*

*3.4 Based on the links – This looks like a Shiny Dashboard. Is this what the authors call EXAM? If so, then EXAM is not a newly created software, it is a visualization platform which reads data from the LifePort machine.*

Yes, EXAM is indeed a Quarto dashboard using the frameworks Shiny (for user interactions) and Plotly (for interactive plots). We also implemented core functions to read, process, and summarize LifePort machine perfusion data (see Table 1). We agree that EXAM is a visualization platform that reads data from the LifePort machine. We have changed the word “software” to “tool”.

To be precise, our “software” has two components: the EXAM dashboard, which takes care of the visualization and user interaction (to upload a data file, select a data file from the dropdown menu), and the data processing (reading raw data, processing and filtering of the time series, and calculating the indicators) which is done by our R package “swt.” This is explained in detail in Table 1.

*3.5 If the above paragraph is correct, then the innovation here is the use of shiny dashboards to visualize the data that has traditionally not been visualized that way.*

Yes; however, as outlined above, this is only one part. The other part is the reading and processing of the raw data files, which could otherwise not be visualized in a dashboard.

*3.6 Then, it is not correct to say there is no free software because what the authors have done is to utilize an existing software (R), which is free, to visualize data.*

Thank you for your feedback. We would like to clarify that while we did utilize the R statistical programming language, which is indeed free software, our contribution involved creating custom functions consisting of hundreds of lines of code. These functions are specifically designed to read, process, and summarize LifePort perfusion raw data, adding significant value and functionality, see lines 238 to 715 in the link below.

<https://github.com/Swisstransplant/swt/blob/main/R/swt.R#L238>

*Data import*

*3.7 Write ASCII in full. Ideally, an acronym needs to be written in full if using it for the first time in the document.*

We replaced the wording “plain text ASCII file” with simply “plain text file”; we believe this is fit for purpose.

Statistical indicators

*3.8 I think it is not correct to say statistical indicators. In other words, are there are other indicators that are not “statistical”? I would suggest, just have the topic to read “Indicators” or “Indicators of interest” or “Outcomes”*

The indicators are based on common statistical summaries (mean, standard deviation), so we believe “statistical indicators” is an appropriate term. However, if the reviewer prefers a different term, we are open to changing it.

Results

*3.9 Table 3: The title talks about median, but the results includes some indicators summarized using mean. What is unclear is, for the indicators summarized using mean, why do we use the 10-90th percentile range? Mean is usually reported alongside standard deviation. Please clarify what is happening here.*

The reviewer spotted a good point, and we are happy to clarify. The indicators are statistical summaries of the time series, e.g., median systolic pressure or mean ice temperature (the time series is summarized across time with one number). The descriptive statistics in Table 3 summarize these indicators across the N=255 kidneys allografts. Thus, 29.4, for example, is the median of all the median systolic pressure values, and 18.8 is the median of all the mean diastolic pressure values; thus all the numbers in Table 3 are medians. We edit the column title to clarify this point.

Then, we used the interdecile range, i.e., the 10th and 90th percentiles as the lower and upper bounds, to display the range of the middle 80% of the data. This approach is more useful for quality control purposes, as it highlights the thresholds for the most extreme 10% of data points on both ends that may require inspection.

*Discussion*

*3.10. This is too brief. I find the discussion to be lacking in depth and width. It is a major shortcoming of this manuscript.*

*I expected the discussion to focus on;*

*a) At least a paragraph on summary of the key findings*

*b) A few paragraphs to discuss what new knowledge or evidence your research has added in the context of other findings in literature. I don’t see any citations in the discussion. I know of papers that have been published that have used RShiny. This is very important. This should be a major part of the discussion.*

*c) A paragraph to discuss any strengths for your research*

*d) A paragraph on the limitations*

*e) Conclusions*

Thank you for this input. We have revised the Discussion section (see our response 2.8) and now include a paragraph about potential limitations. However, we have no key findings and no new knowledge to be discussed in the context of previous studies. The PLOS DH scope states that submissions describing software or other tools are also welcome, and our submission fits perfectly in this category.

*Conclusion:*

*3.11 What is DGF in full? I may have missed it, but as I noted earlier, any acronymn should be written in full if appearing for the first time.*

DGF is delayed graft function (when a kidney recipient requires dialysis the first days post-transplantation). DGF appears throughout the manuscript but is written out in full upon its first appearance in the Introduction section. We did not use the acronym in the Author's Summary or the Abstract.

*3.12 I think the conclusion should focus on what is new, and how can it be scaled up? What policy implications can it have?*

Thank you, we have improved the Conclusion section (see our response 2.9).
